# Role of Opsonophagocytosis in Immune Protection against Malaria

**DOI:** 10.3390/vaccines8020264

**Published:** 2020-05-30

**Authors:** Wolfgang W. Leitner, Megan Haraway, Tony Pierson, Elke S. Bergmann-Leitner

**Affiliations:** 1 Basic Immunology Branch, Division of Allergy, Immunology, and Transplantation/National Institute of Allergy and Infectious Diseases, National Institutes of Health, Bethesda, MD 20852, USA; wleitner@mail.nih.gov; 2 Immunology Core/Malaria Biologics Branch, Walter Reed Army Institute of Research, Silver Spring, MD 20910, USA; megan.r.haraway.ctr@mail.mil (M.H.); tony.pierson.mil@mail.mil (T.P.)

**Keywords:** immune correlates, phagocytosis, serology, complement, exosomes, malaria, circumsporozoite protein, *Plasmodium*

## Abstract

The quest for immune correlates of protection continues to slow vaccine development. To date, only vaccine-induced antibodies have been confirmed as direct immune correlates of protection against a plethora of pathogens. Vaccine immunologists, however, have learned through extensive characterizations of humoral responses that the quantitative assessment of antibody responses alone often fails to correlate with protective immunity or vaccine efficacy. Despite these limitations, the simple measurement of post-vaccination antibody titers remains the most widely used approaches for vaccine evaluation. Developing and performing functional assays to assess the biological activity of pathogen-specific responses continues to gain momentum; integrating serological assessments with functional data will ultimately result in the identification of mechanisms that contribute to protective immunity and will guide vaccine development. One of these functional readouts is phagocytosis of antigenic material tagged by immune molecules such as antibodies and/or complement components. This review summarizes our current understanding of how phagocytosis contributes to immune defense against pathogens, the pathways involved, and defense mechanisms that pathogens have evolved to deal with the threat of phagocytic removal and destruction of pathogens.

## 1. Introduction

The importance of antibodies in the defense against pathogens was recognized a long time ago. Antibodies are currently the only confirmed correlate of protection in vaccine settings where a defined antibody titer can be used to predict protective efficacy of a vaccine [1]. This correlation between antibody titer and protection is, however, strongly dependent on the vaccine formulation. While antibodies are expected to be the main effector molecules after vaccination with a killed or subunit vaccine (e.g., RTS, S, TRAP-ME), life vectors (e.g., irradiated sporozoites or vaccination under chemoprophylaxis) or nucleic acid-based vaccines can be expected to also induce T cell-mediated immunity, although the contribution of cellular immune responses to protection is still poorly understood.

During an immune response, induced via infection or vaccination, antigen-specific B cells are activated by either a T-cell-dependent (TD) or T-cell-independent (TI) pathway. In case of T-cell-dependent activation, B cells first recognize the antigen through their membrane-bound immunoglobulins (mIg; acting as B cell antigen-receptor (BCR)) and subsequently interact with antigen-activated helper T (Th) cells to become fully activated. They subsequently migrate to follicles (B cell compartment of lymphoid organs), which transform into germinal centers after induction of the immune response, and continue to interact with specialized cells such as follicular dendritic cells and follicular T helper cells. These interactions shape the B cell response in terms of isotype switching, affinity maturation, and generation of immunological memory. Driven by affinity to the antigen, B cell clones are selected and then leave the germinal center to become either short-lived plasma cells or memory B cells. An example of a T cell dependent pathway occurs during *Plasmodium* infections, where follicular helper T cells, specifically a CXC chemokine Receptor (CXCR) 5^+^ subset, are necessary to control systemic infections by activating B-cells in germinal centers [2]. Recently, cluster of differentiation (CD)8^+^CXCR5^+^ T cells have been shown to provide B cell help in germinal centers of human lymphoid organs [3].

At the end of the immune response, i.e., after the bulk of the foreign antigen has been cleared and no new antigen is produced, long-lived plasma cells and higher affinity memory B cells are released and subsequently settle in immunological organs, including the bone marrow. Antibodies produced at the beginning of the immune response—compared to those produced at the conclusion of the immune response—usually differ in their affinity to the antigen. Frequently, they also differ in their epitope specificity (“fine specificity”), as well as their isotype. This applies to most protein antigens, which fall into the category of TD antigens. In the case of TI B cell responses, however, the activation of B cells is not dependent on the cell–cell interaction with other cells. TI responses are induced most notably by highly-repetitive antigens (type 1 TI-antigens), which enable the crosslinking of mIg on B cells, regardless of their specificity (mitogenic activity) and have been described as one of many immune escape mechanisms deployed by pathogens. Depending on subsequent signals in the B cells which recognize the antigen, three types of TI-antigens have been described so far, all of which lead to humoral responses that are short-lived, nonproductive (in terms of affinity maturation, isotype switching, and memory induction), and which are thought to drive extrafollicular B cells responses rather than germinal center reactions (reviewed in [4]): (1) Type 1 TI-antigens: the mIg (BCR) is crosslinked in addition to Toll-like receptor (TLR) engagement; the mode of activation is considered mitogenic since the recognition of the antigen leads to polyclonal activation of B cells regardless of antigen specificity. Characteristically, the predominant isotype of a TI-response is IgM. (2) Type 2 TI-antigens: the mIgs (BCR) are crosslinked due to the highly repetitive nature of the antigen, and antigen-specific B cells are fully activated with the help of cytokines produced by accessory cells (such as antigen presenting cells and T helper cells) during the immune response. Type 2 TI-antigens can only activate mature B cells, which in turn may undergo limited affinity maturation [5] and isotype switching to specific isotypes. In contrast, immature B cells are anergized by these types of antigens. An example of a Type 2 TI antigen is the circumsporozoite protein of *Plasmodium* parasites, a major surface protein that displays a highly repetitive central peptide sequence. (3) Type 3 TI-antigens: the mIg (BCR) are crosslinked and B cells are activated after receiving additional signaling through innate cells [4]. Innate immune cells provide costimulation comparable to helper T cells during a T-cell-dependent response and lead to full B cell activation. Currently, it is unknown whether Type 3 TI-antigens can induce affinity maturation or B cell memory responses. However, the main B cell responses are extrafollicular as reported for Type 1 and Type 2 TI-antigens.

While B cells have a wide range of immunological functions, we are restricting this review to the production of antibodies, which act as soluble effector molecules in humoral immune responses. Antibodies can be categorized based on their class and isotype. However, a serological analysis should go beyond these parameters and include the measurement of the antibodies’ affinity to the antigen (expressed as association constant K_A_ in plasmon surface resonance detectors such as the Biacore) and avidity (expressed as dissociation constant K_D_ when measured in Biacore instruments or in chaotropic ELISA assays). Recently, a multiplex (electro-chemiluminescence immune assay) ECLIA-based serological testing platform has been shown to outperform serological ELISA-based assays regarding sensitivity, throughput, and inter-/intra-assay variability and could be used in its place for many applications [6]. An antibody’s affinity is an intrinsic property of the antigen-binding region and typically increases throughout the immune responses and upon re-exposure. Affinity is the consequence of somatic hypermutations that B cell clones induce in the Ig genes to successfully compete with other clones for antigen binding. Avidity is the product of the antibody’s affinity and the valency of the antigen (i.e., the number of antibody binding sites) and the valency of the antibody (i.e., the number of epitope binding sites), which depends on the antibody isotype. IgM, the first soluble Ig to be produced, naturally has a lower affinity than subsequently produced isotypes, which it overcompensates for by forming pentamers. While the binding strength to the antigen may be low to moderate, the high valency (i.e., valency of 10) to the antigen can still sufficiently stabilize the binding between antigen and antibody.

## 2. Classes of Immunoglobulins

Immunoglobulins are categorized based on their heavy chain, and fall into five major classes: IgM, IgD, IgG, IgE, and IgA. This review focuses primarily on IgM and IgG, because neither IgD nor dimeric IgA appear to play a notable role in mediating opsonophagocytosis. IgM is the first immunoglobulin released by newly activated naïve B cells. An exception to this rule is the encounter of TI-1 antigens by B cells, in which case IgM continues to remain the predominant antibody isotype even after repeated exposure. Several parasite-derived antigens have the characteristics of TI-antigens [7]. In the normal serum of a mammalian host, IgG is the dominant isotype and is represented through four subclasses. In humans, these subclasses are IgG1, IgG2, IgG3, and IgG4. B cells receive exogenous signals that determine the switch to one of these subclasses. Cytokines produced by other activated immune cells guide the switch to either cytophilic antibodies (IgG1 and IgG3 in humans), to IgG subclasses that are immunomodulatory (IgG4), or IgG subclasses that activate complement (IgG3, IgG1, and IgG2, listed in order of their potency to activate the complement system) [8]. Cytophilic antibodies mediate uptake of aggregated antigen/pathogen through Fc receptors on macrophages, subpopulations of granulocytes, B cells, and other hematopoietic cells.

The binding of antibodies to Fc receptors triggers either immune cell activation or downregulation, and specific effector function of the cell (oxidative burst, degranulation, phagocytosis). In the context of this review, we are limiting the discussion of the Fc receptors to FcγRI (CD64, binding of IgG3, IgG1), FcγRIIA (CD32; binding all IgG subclasses), FcγRB2 (CD32; binding all IgG subclasses), FcγRIII (CD16, binding all IgG subclasses), FcεRI (binding of IgE), and FcαRI (CD89; binding of IgA). When designing and testing vaccines, it is helpful to understand what functional mechanisms a particular antibody isotype and subclass mediates in order to devise immunization strategies that promote the induction of those antibody classes capable of inducing those immune mechanisms that are most effective against a particular pathogen. The preferential induction of antibody classes can be achieved by choosing specific vaccine adjuvants, vaccine platforms, or by modulating vaccination regimens. While the modulation of immune responses through these approaches is conceptually very appealing and may appear straight-forward, it is hampered by a fundamental lack of understanding of what antibody-mediated immune mechanisms are most effective against a specific pathogen.

Although the analysis of antibody responses induced by natural infection can provide some insights, one must consider the potential effects of immune escape mechanisms of the pathogen [9]. These may result in the targeting of epitopes by the host that disrupt antibody maturation, the preferential induction of antibodies to decoy epitopes (e.g., TI antigens), or the induction of Ig subclasses, which bind to inhibitory Fc receptors, thus interfering with phagocyte activation. Using epitopes to disrupt antibody maturation has been described for the *Plasmodium* parasite. Many of *Plasmodium’s* antigens have repeated sequences that encode epitopes, and cross-reactions among epitopes with related sequences can cause mutated B cells, which results in abnormal antibody maturation and thus impairment of the development of protection in the host [10]. On the other hand, we recently described a scenario in which interference with phagocytosis of opsonized pathogens provided protection from infection. In this case, vaccine-, and possibly also infection-mediated induction of antibody subclasses, which promote phagocytosis, are unexpectedly associated with loss of protection from malaria infection, while the presence of IgG4, which interferes with the activity of IgG1 and IgG3 by preventing phagocytosis, is associated with sterile protection in vaccinated human volunteers [11,12]. This finding underscores the importance of comprehensively analyzing antibody responses to understand the functional consequence of specific isotypes and subclasses, rather than simply analyzing total antibody titers.

In other disease models, studying natural immunity to a pathogen often reveals protective immune mechanisms. The hallmarks of natural immunity to malaria are the continued presence of blood-stage parasites, but the absence of severe symptoms, and the short duration of this form of immunity in the absence of continued exposure to infectious bites. In contrast, the goal for a vaccine is to induce long-lived, sterilizing immunity without the need for frequent boosting. It is, therefore, not surprising that the immune mechanisms induced by natural immunity vs. vaccination are quite distinct including the specificity of the immune response (which life stage, antigen, or epitope is targeted), the class of B cells that are activated by the immunogens, and the biological functions of the resulting antibodies.

## 3. Fc Receptors

The efficiency of antibody binding to Fc receptors depends on isotypes and what immune cell the antibody binds to since different immune cells express distinct subsets of Fc receptors. This review focuses on the importance of FcRs for phagocytosis and cell regulation, namely FcαRI (CD89), FcεRI, FcγRI (CD64), FcγRII (CD32), and FcγRIII (CD16). Binding of immune complexes to FcγR has various consequences: activation (or inhibition of activation) of cells, secretion of pre- (or anti)-inflammatory factors [13,14,15,16], phagocytosis, cellular activation and maturation of dendritic cells and in efficient MHC class II-restricted presentation of the exogenous peptides [17].

The interaction with Fc receptors is not solely governed by the heavy chain (i.e., the isotype) of the immunoglobulin, but also its glycosylation pattern [18,19]. Vaccine adjuvants can change glycosylation patterns and thereby modulate immune responses since changes in glycosylation can lead to the binding of an antibody to an activating rather than an inhibitory Fc receptor and vice versa [20]. Vaccine adjuvants can be used to drive Th profiles of vaccine-induced immune response and promote induction of specific isotypes, in addition to simply enhancing the response (reviewed in [21]). Matching of an adjuvant with a particular vaccine to induce the most efficacious isotype or IgG subclass is not being implemented yet, mostly because of, as discussed above, a lack of knowledge of what constitutes a “desirable” response against most pathogens. It may, however, be possible to gain insights into the role of antibody subclasses by adoptively transferring isolated subclasses into naïve hosts followed by a pathogen challenge. While such an approach is technically relatively straight-forward, the epitope specificity of antibodies with different Fc portions is a major caveat since it may differ between subclasses. Such differences would make it difficult to interpret the results obtained with isolated antibody subclasses.

In addition to the presence of isotypes and antibody subclasses within isotypes, allelic variations among IgG subclasses (allotypes) have been described between individuals within an ethnic group and between ethnic groups in general [22,23]. These variations add another layer of complexity when analyzing and interpreting immune responses in different individuals and may be one of the many factors (which include, e.g., differences in the microbiome, chronic infections, HLA, nutritional status) that contribute to the difficulty of transferring vaccination results obtained in volunteers in developed countries to vaccine recipients in developing nations.

## 4. Biological Consequence of Antibody Binding to Antigen

An essential biological function of antibodies is their ability to activate factors such as the complement system or cells after binding to Fc receptors, which prompts the immune cell to display its effector functions, such as phagocytosis (by macrophages, neutrophils) or degranulation (by granulocytes, mast cells, platelets).

Neutrophils are the most abundant leukocytes that form the first line of defense against malaria (reviewed in [24]). Not surprisingly, various mosquito salivary factors and parasite-derived factors aim to interfere with neutrophil function, thus facilitating infection after a mosquito bite [24]. The antiplasmodial functions of neutrophils include phagocytosis, production of reactive oxygen species (ROS), and neutrophil extracellular traps (NETs). The frequency of neutrophils increases dramatically during acute malaria infections, and the prevalence of malaria pigment-containing neutrophils is used to stage the severity of disease [25]. Neutrophils are highly efficient in phagocytizing opsonized antigens; however, the binding of antibodies to their pathogen target is not necessarily beneficial to the host, and depending on the outcome of this interaction, it is necessary to distinguish between antagonistic and agonistic antibodies. Both exert their beneficial vs. detrimental effects through different pathways, which have been described in a variety of pathogen systems.

### 4.1. Antagonistic Antibodies

#### 4.1.1. Neutralization of the Pathogen by Interfering with the Binding to Host Cells and Preventing the Further Spread of the Infection 

The threshold of neutralizing antibody (nAb) titers Is known for a variety of bacterial and viral diseases (reviewed in [1]) Antibodies play a significant role in the immune response against parasites, and in the case of malaria, neutralizing antibodies can target each of the malaria-specific life stages, i.e., sporozoites, merozoites released from ruptured liver, or blood schizonts, as well as sexual stages. Neutralizing antibodies, which recognize sporozoites shortly after they had been delivered by a mosquito, would be expected to have the highest chance of success in eliminating disease simply based on parasite numbers: during an infectious blood meal, less than 300 sporozoites are introduced into the host. However, the neutralization must be virtually perfect since any surviving parasites might still be able to establish a blood infection. In contrast, the neutralization of blood-stage parasites (merozoites) would at least reduce the burden of parasitemia and, thus, the severity of disease. Only if titers are sufficiently high can neutralizing antibodies to blood-stage antigens abrogate the infection. Neutralization of malaria parasites can be achieved through either agglutinating the merozoites, or preventing the rupture of mature erythrocytes and, therefore, the release of new parasites [26]. Targeting sexual stages by a transmission-blocking vaccine does not benefit the host directly, but is designed to prevent the infection of mosquitoes after a blood meal and, thus, the further transmission of parasites (altruistic vaccine). Transmission-blocking antibodies have been described as complement-fixing [27], but may also work by blocking key parasite surface molecules after a blood meal and inside a mosquito’s midgut.

Growth inhibition displayed by antimalaria antibodies is evidenced after successful invasion of the host cell (hepatocyte, erythrocyte); while growth inhibitory antibodies cannot prevent infection, they remain bound to the pathogen and interfere with the establishment of infection. One example for such activity has been reported for antibodies targeting the merozoites surface protein (MSP)-1, which has been a leading antigen candidate for blood-stage vaccines to date [28]. MSP-1 specific antibodies display a variety of biological effects on the blood-stage parasite [29]. While some of the antibodies prevent invasion of new erythrocytes through various pathways, other antibodies inhibit the growth of the intraerythrocytic parasite [29]. Growth inhibition is thought to be mediated by blocking the function of the MSP-1 protein, which is crucial for the formation of the food vacuole [30,31].

Another protein on the surface of *Plasmodium* merozoites has been of great interest as a target for blood-stage vaccines. PfRH5, located on the apical end of merozoites, is a member of the reticulocyte-binding protein family and plays a role in merozoite invasion. It has been reported that when using vaccine induced IgG to neutralize 3D7 parasites, there was strong growth inhibition of the parasite, and it was shown to be more successful when compared against other antigens, including MSP-1. Antibodies against PfRH5 were also effective when tested on other parasite strains such as FVO, Dd2, GB4, and CAMP, and were thus deemed to have cross-strain neutralizing ability [28,32].

#### 4.1.2. Mediation of Opsonization/Phagocytosis

Opsonization/phagocytosis is the binding of antibodies to a pathogen to mediate uptake by phagocytic cells or binding of complement factors resulting in the destruction of the pathogen by either complement-mediated lysis or binding to complement receptors immune cells triggering uptake and activation of these cells. Antibodies displaying this biological function are often being referred to as non-neutralizing, anti-pathogen antibodies [33].

Antibody-mediated complement-dependent (Ab-C’) inhibition is an activity of naturally acquired human antimalarial antibodies that inhibits merozoite invasion of red blood cells during blood-stage malaria infections. C1q fixation has been found to be the main mediator of Ab-C’ inhibition, and the main targets are *Plasmodium* surface proteins MSP-1 and MSP-2 [34]. Even though the complement system is a main line of defense of the human immune system, it has been found that *Plasmodium* has also accordingly acquired immune-escape strategies via another surface protein, Pf92. This protein can actively recruit a host regulatory factor, FH (Factor H), when exposed to human serum. FH is a human complement regulator that protects tissues from atypical complement activation, and the parasite uses this mechanism as a way to ensure protection during blood-stage infections and escape complement-mediated lysis [35].

Phagocytosis of opsonized targets is an important immune defense mechanism against parasites, such as *Plasmodium*, but is inevitably also the target of pathogen-escape mechanisms. A study in 1990 focused on the biological consequence of antibody binding to sporozoites and interaction of these immune complexes with immune cells and hepatic cell populations [36]. Based on this report, sporozoites can, at least in vitro, evade phagocytic cells upon phagocytosis, a process which results in the lysis of these phagocytes. We previously investigated the role of antibodies induced by the RTS,S malaria vaccine in mediating phagocytosis and the role of this biological activity in protective immunity [11] (Section 5.3). Although vaccine-induced antibodies were capable of mediating phagocytosis, the extent of phagocytosis was negatively correlated with protection. While others had previously assumed that inducing IgG subtypes that mediate phagocytosis might be associated with protective immunity, we are currently pursuing the hypothesis that high-avidity subclasses, such as IgG4, which are increased in individuals with sterile protection and which do not mediate phagocytosis, outcompete phagocytosis-mediating antibodies [12]. Our findings indicate that a lower level of phagocytosis is more beneficial to the vaccine recipients, and suggest that, in the case of malaria, phagocytosis of sporozoites is part of the pathogen’s escape mechanism rather than a host-protective mechanism. Additional functional studies on the RTS,S induced antibodies and their role in protection revealed a range of activities including complement binding and other, nonphagocytic, Fc-mediated functions [37].

The role of phagocytosis in combating the later, i.e., erythrocytic and asexual, stages of malaria was established by studying serological responses in different age groups of individuals with natural immunity [38,39]. Opsonizing antibodies against parasites at these stages are the consequence of repeated infections. Their titers increase with age and correlate with protection against high-density parasitemia in the blood and clinical disease [39]. The positive correlation between opsonization levels of merozoites and exposure, as well as protection was confirmed independently [40]. It does, however, seem difficult to reconcile these results with studies described below that report how the phagocytosis of blood-stage malaria parasites interferes with the functionality of the phagocyte. However, these two processes—the induction of opsonizing antibodies, which triggers the removal of parasites, and the downregulation of phagocyte activity, including phagocytosis after the uptake of the parasite—should not be viewed in isolation, but in light of the fact that the parasite’s goal is the induction of a chronic infection (“natural immunity”). Such a state represents a delicate balance between a) limiting the parasite density through protective immune mechanisms, which guard the host against morbidity and mortality while not clearing the pathogen, and b) immunosuppression by the pathogen, which must not be too efficient since a severely immunocompromised host would be highly susceptible to other pathogens and only available as a host for the parasite for a short period of time.

The impact of phagocytosis of mature sexual erythrocytic stages (gametocytes) has been less well studied. One study demonstrated that gametocytes are not subject to phagocytosis, and this allows the unimpeded transmission of the parasite to the insect host during a blood meal [41]. Phagocytosis of gametocytes also does not play a significant role in naturally acquired immunity [42].

### 4.2. Agonistic Antibodies

Born out of a need to counter the attack by the immune system, pathogens have developed a series of immune escape mechanisms [43]. We summarize here the mechanisms that involve the exploitation or modulation of humoral responses of the infected host as antibody-dependent enhancement (ADE) of infections.

Studies on *Plasmodium* have shown that antibodies can assist the malaria parasite at different life stages in the infection of host cells: (1) antibodies to the sporozoites can increase infectivity and development of liver stage parasites [44,45,46]; (2) antibodies to parasite antigens expressed on merozoites can facilitate the infection of red blood cells either by themselves or in collaboration with complement [47,48]. Studies investigating the role of growth-inhibitory antibodies in the field have revealed an interference between malaria-specific antibodies which can result in the loss of parasite-inhibiting functions [49]. The interference is likely due to “blocking” antibodies preventing inhibitory antibodies from binding to either a crucial antigen [50] or crucial epitope. The latter was reported for the merozoite surface protein (MSP-1) where the fine specificity had a major impact on the biological function induced by the respective antibodies [50,51,52]. MSP-1 is a 195 kD protein that constitutes the major coat protein of the blood stage merozoite. It undergoes sequential autoproteolytic cleavage resulting in a 42 kD fragment (MSP-1p42) that remains on the surface of the merozoite and plays a major role at the time of red blood cell invasion. MSP-1p42 has two epidermal growth factor (EGF)-like domains at the C-terminus that have been shown to be targeted by either blocking or inhibitory monoclonal antibodies [52]. When performing serial depletions of C-terminal antibodies from malaria-endemic sera specific for the EGF-like domain I vs. EGF-like domain II, it was demonstrated that natural exposure induces blocking antibodies that interfere with the antagonistic effect [51], and the epitopes for such blocking antibodies are located in EGF-like domain I [50,52].

### 4.3. Autoreactive Antibodies

These Abs are induced in the course of an immune response and, due to molecular mimicry, mediate the destruction of healthy cells and tissues (reviewed in [53,54]. There are numerous reports that anti-red blood cell (RBC) antibodies induced by malaria can lead to severe anemia [55]. These autoreactive antibodies have numerous targets including single- and double-stranded DNA, RNA, phospholipids, phosphatidylserine (PS), smooth muscles (reviewed in [54]. Recently, the marked increase in the frequency of atypical (Fc-Receptor (FcR)5^+^ T-bet^+^) B cells producing anti-PS antibodies that contribute to anemia has been reported [56]. Malaria infections appear to regulate the expansion of B cells expressing the inherently autoreactive VH4-34 H chain in residents of malaria-endemic area in West Africa. While genetically there was no difference in the overall frequency of VH4-34H^+^ B cells in peripheral blood between residents in West Africa and the U.S, there was a higher proportion of memory B cells with that specificity after acute febrile malaria [57].

### 4.4. Tagging Antibodies

Such antibodies bind to their target, but do not exhibit any obvious biological function that can be detected in currently available functional assays. While the magnitude of an antibody response can be measured by serological assays, only a functional assay would be able to determine the consequence of antibody binding to the target. It is possible that the binding of such tagging antibodies may obstruct the binding of other functional antibodies thereby modulating the biologic consequence.

## 5. Phagocytosis

Phagocytosis may be considered as one of the most important anti-pathogen activities besides the direct neutralization of pathogens by antibodies. Even neutralized pathogens need to be removed, and the retention of immune complexes has been shown to contribute to the maintenance of immunological memory by providing a persistent source of antigen that is used for the restimulation of memory lymphocytes in the absence of re-exposure to the pathogen. Several pathways that mediate phagocytosis have been described in the literature: (1) Binding of pathogen-associated molecular patterns (PAMPs) to pattern recognition receptors (PRR) such as TLRs or scavenger receptors [58]; while PAMP-mediated binding to phagocytic cells directly triggers the uptake into the cells, opsonized (i.e., complement and/or antibody coated) pathogens bind to either complement receptors or Fc receptors and trigger phagocytosis. (2) Recognition of PAMPs by complement factors mannose binding lectin (MBL), C1q or C3b; the deposition of these “sentinel” factors activate the complement cascade, which results in the lysis of the pathogen. Cellular complement receptors (CD35, CD21, CD11b/CD18, CD11c/CD18) not only mediate binding and uptake of complement-fixed antigens, but also act as coreceptors (particularly CD21 [59]) to activate immune cells. (3) Binding of antibodies (induced by either natural or vaccine immune responses) to the pathogen with subsequent complement deposition and lysis of pathogen or Fc-receptor-mediated interactions with immune cells.

### 5.1. Escaping Immune Recognition and Phagocytosis as a Means of Pathogen Survival

Since these mechanisms and host molecules pose a significant threat to the pathogen, several immune escape mechanisms have evolved (reviewed in [36]). The following represent some of the most prominent, but certainly not all of these escape mechanisms: (1) Interference with complement activation after PAMP recognition [53,60]; (2) downregulation of PAMPs or expression of complement inhibitors [60]; (3) interference with the recruitment of phagocytic cells to infection site through secretion of chemokine-modulatory proteins [61,62]; (4) expression of factors which prevent or cleave cell adhesion molecules crucial for phagocytosis [63]; (5) prevention of phagocyte activation. An example is the stimulation of IL-4 release by *Plasmodium* parasites, a cytokine which suppresses macrophage activity [64]; (6) pathogens have developed strategies to evade phagocytosis by signaling through FcγRIII (CD16) [65].

When first encountering *Plasmodium falciparum*, macrophages can kill the parasite through the release of cytotoxic factors in the absence of antibodies. The interaction of immune cells with sporozoites leads mainly to tolerization of the immune response [66]. Similarly, malaria pigment (hemozoin), the degradation product of the infected RBC’s hemoglobin, has been shown to irreversibly inhibit macrophages after phagocytosis [67]. However, phagocytosis in many of the affected phagocytes appears to be significantly reduced, not entirely prevented, since the latter would leave the infected host highly susceptible to other infections, which is not in the interest of the parasite.

As indicated above, various pathogens have adopted a “Trojan horse” strategy whereby they reside in the phagocyte, protected from clearance by immune mechanisms. While inside the phagocyte host and carrier, these pathogens deploy a battery of strategies to modulate both innate and adaptive immune responses [68]. *Plasmodium*, apart from selecting host tissues and cells that are somewhat immunoprivileged (namely erythrocytes) or immune-dampening (in case of the liver) resides within vacuoles of its mammalian host cells; the intrahepatic parasite lives in a vacuole, which limits the leakage of parasite-derived proteins/peptides into the host cytoplasm. This leakage of antigens would normally result in immune recognition of the infected cell by cytotoxic T cells and/or natural killer cells. The intraerythrocytic parasite also resides within a parasitophorous vacuole, an even safer host cells since erythrocytes do not express immune surveillance molecules such as the major histocompatibility complex class I, which is present on most other mammalian cells. The parasite-derived antigens that are expressed on the erythrocytes play a crucial role in sequestration and preparation for the next round of invasion (resetting); to avoid neutralizing antibodies, the parasite displays a high rate of polymorphism within regions of these antigens that do not have a functional role. Furthermore, the parasite expresses antigens that mainly have immune decoy functions. One of the antigens involved in immune decoy is *P.falciparum* Erythrocyte Membrane Protein (PfEMP-1), an antigen expressed on the surface of infected red blood cells that mediates rosette formation [69]. Rosette formation is achieved by binding of malaria-specific IgM antibodies to infected red blood cells; Rosette formation prevents complement activation and the resulting cellular aggregates recruit uninfected red blood cells, thereby facilitating subsequent infection [69].

### 5.2. Hijacking Phagocytosis by Parasitic Protozoa

Phagocytosis is clearly beneficial in the defense against pathogens, and numerous reports have described the involvement of phagocytosis in the response against protozoan parasites. [70]. Once inside the phagocyte, the parasite has been shown to interfere with signaling involved in the cellular activation of phagocytes, which prevents its destruction, but also interferes with the ability of the phagocyte to induce an adaptive immune response [71].

The phagocytosis of malaria parasites has been shown to be a double-edged sword: a large proportion of sporozoites are eliminated by phagocytosis before reaching their final target, hepatocytes [72]; however, the entry of sporozoites into phagocytes is also an important step in the infectious process and particularly Kupffer cells in the liver are hijacked by invading sporozoites [36,73]. When arriving in the liver, sporozoites are initially arrested in the sinusoid. At this anatomical site, they seek Kupffer cells, which they invade in a process that allows the parasite to enter the liver parenchyma [73]. Unlike in the case of classical phagocytosis, the process by which sporozoites penetrate Kupffer cells (and potentially also endothelial cells that are part of the sinusoid barrier) can involve multiple passages of the parasite through the phagocyte.

Sporozoite cell traversal involves rupture of the host cell membrane. It has been discovered that SPECT (sporozoite micronemal protein essential for cell traversal) is essential for the initial steps of hepatocyte traversal for the parasite. SPECT contains a MACPF (membrane attack complex/perforin)-related gene domain that is known to be a pore-forming protein that creates channels in the cell membrane and may be a mechanism of entry for the sporozoite into the host cell [74]. Another protein that is crucial in the traversal of the parasite through host cells in both the insect and the mammalian host, is the cell traversal protein for ookinetes and sporozoites (CelTOS), a promising new vaccine candidate that has shown protective efficacy in preclinical models and is currently being evaluated in Phase I clinical trials [75,76]. How antibodies against CelTOS mediate protection is not clear yet, but it is conceivable that either blocking antibodies or opsonizing antibodies could interfere with a successful infection by preventing the migration of sporozoites to their target cells, or by routing them to the phagosome while interfering with the parasite’s ability to control its fate inside a phagocyte, respectively. The repeated exposure to an otherwise highly effective phagocyte with an arsenal of antimicrobial mechanisms, surprisingly, does not harm the malaria parasite, which has prompted the search for the underlying escape mechanism: normally, the binding of pathogen-associated molecules to receptors on the surface of phagocytes results in the activation of the immune cell, the subsequent phagocytic uptake of the tagged pathogen, and the ultimate destruction of the pathogen. The interaction of the parasite’s major surface antigen (the circumsporozoite protein, CSP) with LRP-1 (low-density lipoprotein receptor-related protein) and proteoglycans on the surface of Kupffer cells instead triggers cellular signals that prevent the induction of an oxidative burst response [77]. Furthermore, rather than triggering the release of inflammatory cytokines, this interaction with a sporozoite prompts the phagocytic cell to switch its cytokine profile to an inhibitory phenotype (based on IL-10) [78].

Even during the progression from liver-stage infection to a blood-stage infection, the malaria parasite continues to manipulate their host cells. To reach erythrocytes, the hepatic merozoite must pass through the liver Space of Disse and the sinusoid endothelium. The merozoites bud off from the hepatocyte into vesicles known as merosomes, which are covered in host-cell-derived membranes, allowing *Plasmodium* to mask itself from the host immune system. The merosomes enter into the pulmonary capillaries and disintegrate to release the merozoites, which proceed in establishing blood-stage infections [79,80].

Once the malaria infection has progressed to a blood-stage infection, phagocytic cells continue to be manipulated by the parasite. Phagocytosis of infected RBCs was shown to have detrimental effects on the subsequent functional activity of the macrophages, dramatically reducing their phagocytic activity [67]. The key molecule in this process is hemozoin, which has also been shown to interfere with the activation and maturation of phagocytic cells [81], resulting in apoptosis of the phagocyte. Interference with phagocyte function inevitably affects antigen presentation. Together with the downregulation of costimulatory molecules [82], this interference with phagocyte function negatively impacts the induction of adaptive immune responses [83], which enables the establishment of a long-term infected state. While infected RBCs are expected to mostly interact with splenic phagocytes (and here, particularly macrophages in the red pulp of the spleen), the breakdown of the blood–brain barrier in case of cerebral malaria also exposes microglia to the parasite. The uptake of extracellular vesicles triggers a neuroinflammatory response in microglia, leading to an increase in Tumor Necrosis Factor (TNF)-α (inflammatory cytokine) expression and a decrease in IL-10 (immune suppressor) expression [84]. As a result, microglia-triggered neuroinflammatory responses likely exacerbate disease [85].

The modulation of the functional activity of phagocytic cells and their signaling is not restricted to immune cells. A study monitoring development of the parasite in the liver over extended time demonstrated that infected hepatocytes recognize the parasite in its parasitophorous vacuole as measured by a panel of autophagy marker proteins. Most of the parasites, however, survived this initial attack, which was followed by a gradual loss of the autophagy markers and associated lysosomes and the successful establishment of a liver-stage infection [86].

### 5.3. Phagocytosis as a Functional Readout Method

Various phagocytosis assays have been described in the literature, but many are not suitable as standard assays for one reason or the other. A major shortcoming of many protocols is the requirement for components that are not easily obtainable. For example, phagocytosis assays described for analyzing malaria-immune sera call for fresh/cryopreserved malaria parasites [87] or primary human phagocytes [67]. The use of fresh sporozoites is not feasible for many laboratories or are rather costly (in case of cryopreserved sporozoites). Moreover, the involvement of parasites or primary cells also introduces significant assay-to-assay and laboratory-to-laboratory variations. To be able to evaluate opsonophagocytosis with simple reagents and in a highly reproducible manner, we have developed an improved, ultrasensitive, high-throughput assay [11]. This assay allows the rapid assessment of the functional activity of malaria-specific antibodies, but can also be applied to other pathogen systems. The assay can be performed in any laboratory equipped with a basic flow cytometer, eliminating the need for malaria parasites or microscopy. Commercially available neutravidin-coated microspheres with a size of 1 µm are incubated with biotinylated recombinant malaria antigen and are used as a substitute for malaria parasites. The beads are incubated with antisera to allow opsonization and then added to either freshly isolated murine peritoneal macrophages or monocytic cell lines such as IC21 (mouse) or THP-1 (human). The extent of phagocytic uptake of beads by the phagocytic cells is measured by flow cytometry, and both the number of cells that have ingested beads as well as the mean fluorescence intensity are recorded. We have analyzed the functional activity of vaccine-induced antibodies using this method and made the surprising observation that individuals protected after vaccination had a lower phagocytic index than individuals that were not protected in a controlled human malaria challenge [11]. This was the first report of the inverse relationship between this important biological function and protection from malaria infection.

Since the assay is not technically demanding, we anticipate that many laboratories around the world will be able to adopt the method and characterize immune response induced either by various vaccine platforms or after natural exposure to pathogens. We hope that these efforts will help decipher the role of phagocytosis in the protective immune response against various pathogens. The analysis of antibody subtypes and their role in opsonophagocytosis can also help in designing and refining immunization strategies and vaccine formulations. The results provide feedback regarding what functional activity of the antibodies (i.e., enhance or prevent phagocytosis) is associated with protection.

## 6. Outlook

The coevolution of host and pathogen has led to complex interactions and mechanisms by which the immune system can successfully defeat the pathogen, while the pathogen has simultaneously learned how to exploit the host’s defense strategies in an effort to infect and spread. The assessment of the functional activity of antibodies provides insight into the types of immune responses that are induced by vaccination or infection, and is superior to the simple quantitative measurements of antibody titers. Nevertheless, one has to keep in mind that such functional activities of the host’s immune system can be exploited by the pathogen to escape immune recognition and destruction. This is made even more complicated when the effects of host- or parasite-derived extracellular vesicles are considered. The increasing efforts to evaluate the biological activity of immune mechanisms will lead to the identification of mechanisms which mediate immune protection and/or immune escape.
